# Mrs. Browning’s Warning

**Published:** 1888-08

**Authors:** 


### MRS. BROWNING'S WARNING.

Mrs. Joseph Browning, a beautiful and accomplished young woman, the wife of a railroad engineer well known in this city, who for some time past has been running an engine on the Wabash, St. Louis and Pacific between St. Louis and Moberly, Mo., retired to her room at No. 1911 Izard street last night, and in a few moments was enjoying the pleasant slumber which the young only appreciate. Her mother, Mrs. P. H. Roche, who was here with her visiting her father, who resides at the number designated above, soon afterwards joined her. About 11 o'clock the latter was awakened by hearing her daughter cry out in a tone of terror, "Joe! O Joe!" and at the same time spring out of bed. Mrs. Roche at once arose and said, "Lula, What is the matter?" to which the young wife replied, "O mamma, something terrible is going to happen. Just now Joe came and stood by the bedside, and his face was pale and sad, and I know he is dead." Mrs. Roche tried to soothe her by remarking she was suffering from nervous trouble, but to no purpose, and only a moment afterwards when she went to lie down, she again exclaimed, "There, there he is now in the doorway." Mrs. Roche again assured her that it was only a phantasy, but to no purpose, and the mother and daughter again lay down, but not to sleep, for by this time both their imaginations had been wrought up to the highest pitch.

At 1:20 this morning a knock came to the door, and in response to the inquiry of "Who is there?" the reply came "Telegram." Upon opening the message it was found to be for Philip Roche, a brother of Mrs. Browning, who lives at 1209 Douglas street, and read as follows.

"Moberly, Mo., July 14.—Philip Roche, Omaha: Joe died at 11 o'clock. Come on first train and bring Lula. ED. CHAMBERLAIN."

The scene which followed beggars description. Mrs. Browning, who, by the way, is soon to become a mother, was frantic with grief, and the united efforts of the family and neighbors were required to keep her under control. Only the assurance that if she did not calm herself she would not live to go to Moberly to-day had the desired effect upon her. A physician was summoned and opiates administered, and she was finally quieted. Mr. Philip Roche, the brother, came up soon, and at 3:15 yesterday the family left for Moberly on the Wabash.

The case is indeed a sad one, the couple having been married but a year. Browning's death was caused by falling from his engine July 3. At the time he did not suppose he was hurt badly and took his run the next day as usual. Two days ago he took to his bed and last night died. It is supposed he was injured internally. The strange dream of Mrs. Browning is hard to account for, but the facts are as stated, and the case goes to show that a chain of thought existing between human minds can in extreme cases be made to assert itself, no matter how great the distance between the persons may be.—*Omaha Bee.*

